# Radiomics and Machine Learning for Radiotherapy in Head and Neck Cancers

**DOI:** 10.3389/fonc.2019.00174

**Published:** 2019-03-27

**Authors:** Paul Giraud, Philippe Giraud, Anne Gasnier, Radouane El Ayachy, Sarah Kreps, Jean-Philippe Foy, Catherine Durdux, Florence Huguet, Anita Burgun, Jean-Emmanuel Bibault

**Affiliations:** ^1^Radiation Oncology Department, Georges Pompidou European Hospital, Assistance Publique-Hôpitaux de Paris, Paris Descartes University, Paris Sorbonne Cité, Paris, France; ^2^Cancer Research and Personalized Medicine-Integrated Cancer Research Center (SIRIC), Georges Pompidou European Hospital, Assistance Publique-Hôitaux de Paris, Paris Descartes University, Paris Sorbonne Cité, Paris, France; ^3^Department of Oral and Maxillo-Facial Surgery, Sorbonne University, Pitié-Salpêtriére Hospital, Paris, France; ^4^Univ Lyon, Université Claude Bernard Lyon 1, INSERM 1052, CNRS 5286, Centre Léon Bérard, Centre de Recherche en Cancérologie de Lyon, Lyon, France; ^5^Department of Radiation Oncology, Tenon University Hospital, Hôpitaux Universitaires Est Parisien, Sorbonne University Medical Faculty, Paris, France; ^6^INSERM UMR 1138 Team 22: Information Sciences to support Personalized Medicine, Paris Descartes University, Sorbonne Paris Cité, Paris, France

**Keywords:** radiomics, machine learning in head and neck cancer, predictive medicine, radiation oncology, treatment planning

## Abstract

**Introduction:** An increasing number of parameters can be considered when making decisions in oncology. Tumor characteristics can also be extracted from imaging through the use of radiomics and add to this wealth of clinical data. Machine learning can encompass these parameters and thus enhance clinical decision as well as radiotherapy workflow.

**Methods:** We performed a description of machine learning applications at each step of treatment by radiotherapy in head and neck cancers. We then performed a systematic review on radiomics and machine learning outcome prediction models in head and neck cancers.

**Results:** Machine Learning has several promising applications in treatment planning with automatic organ at risk delineation improvements and adaptative radiotherapy workflow automation. It may also provide new approaches for Normal Tissue Complication Probability models. Radiomics may provide additional data on tumors for improved machine learning powered predictive models, not only on survival, but also on risk of distant metastasis, in field recurrence, HPV status and extra nodal spread. However, most studies provide preliminary data requiring further validation.

**Conclusion:** Promising perspectives arise from machine learning applications and radiomics based models, yet further data are necessary for their implementation in daily care.

## Introduction

Machine Learning (ML) has promising applications in radiation oncology. This technique can process a high number of heterogeneous parameters and its recent development has led to a new paradigm in predictive medicine with hope for improved prognosis classification and toxicity prediction for a better treatment strategy. ML algorithms are all the more powerful that they include data from many different sources that can be clinical, biological, genomic and radiologic. Radiomics are actively being explored as prognostic and predictive tools, correlated with histologic and genomic characteristics in several cancer types (1–3). ML techniques may also enhance the radiation oncology workflow management thanks to automation of treatment planning steps such as automatic delineation or adaptive radiotherapy.

### Radiomics

Innovation in medical imaging has led to a dramatic increase of resolution and imaging modalities (MRI, PET TDM, CT), imaging agents, reproducible protocols, and image analysis such as radiomics. Radiomics consists in extracting hundreds of quantitative features by an automated or semi-automated software. It relies on the hypothesis that mineable data can be extracted from medical images and provide additional information on gene protein and tumor phenotype and then used for patient care (4, 5).

Contrary to subjective evaluation of tumor characteristics such as necrosis or heterogeneity, radiomics use data characterization algorithms, automatically extracted from a delineated volume/region of interest (on a CT, PET, or MRI scan), to render a mineable feature space. They can be classified into the following categories (6).

- First order features describing the distribution of voxel intensities (including intensity features from wavelet decompositions of the original image).
- Shape features: related to the shape of the volume.
- Texture features: revealing intra-tumoral heterogeneity differences (including texture features from wavelet decompositions of the original image).

**Figure 1:**
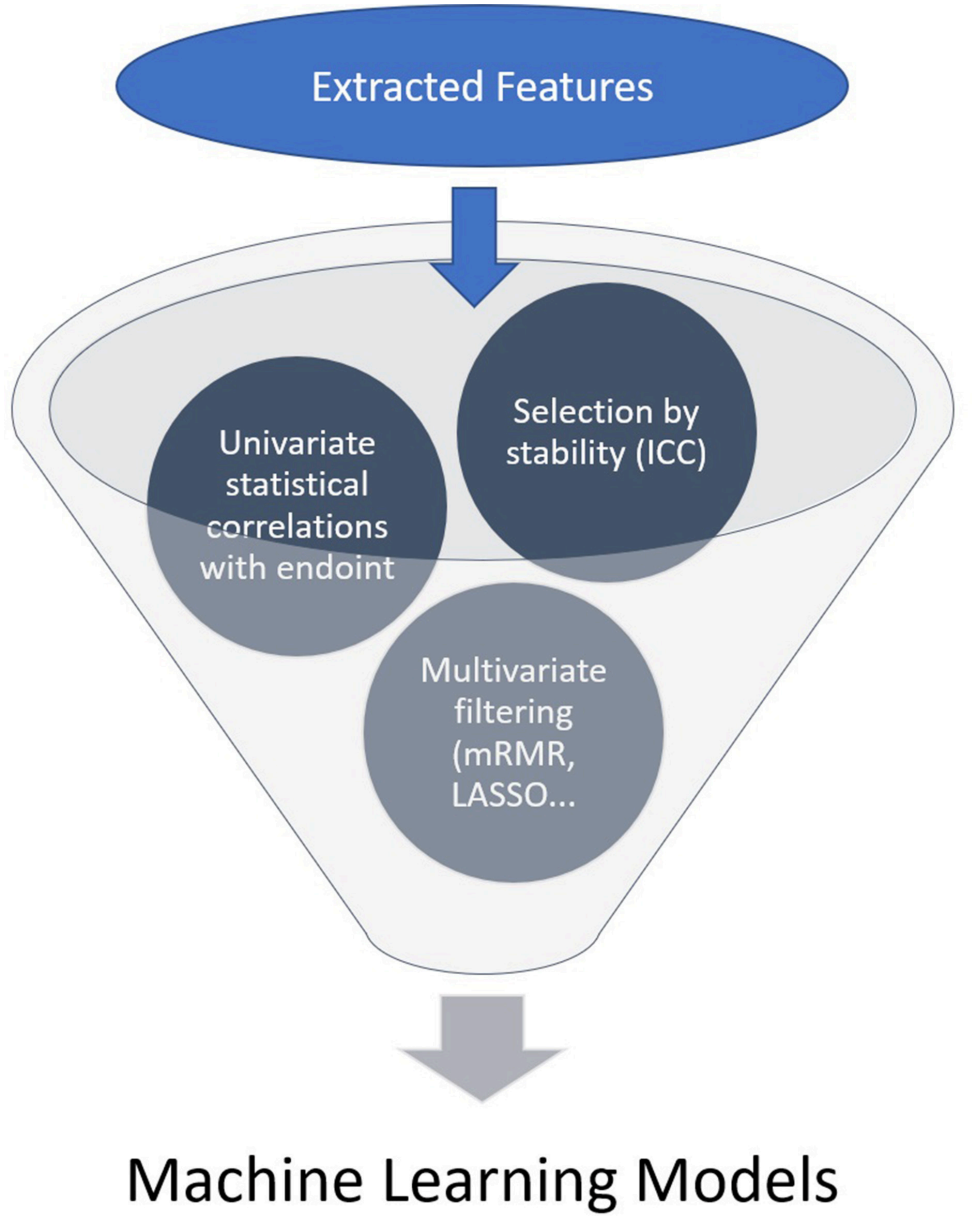
Feature selection general process.

**Figure 2:**
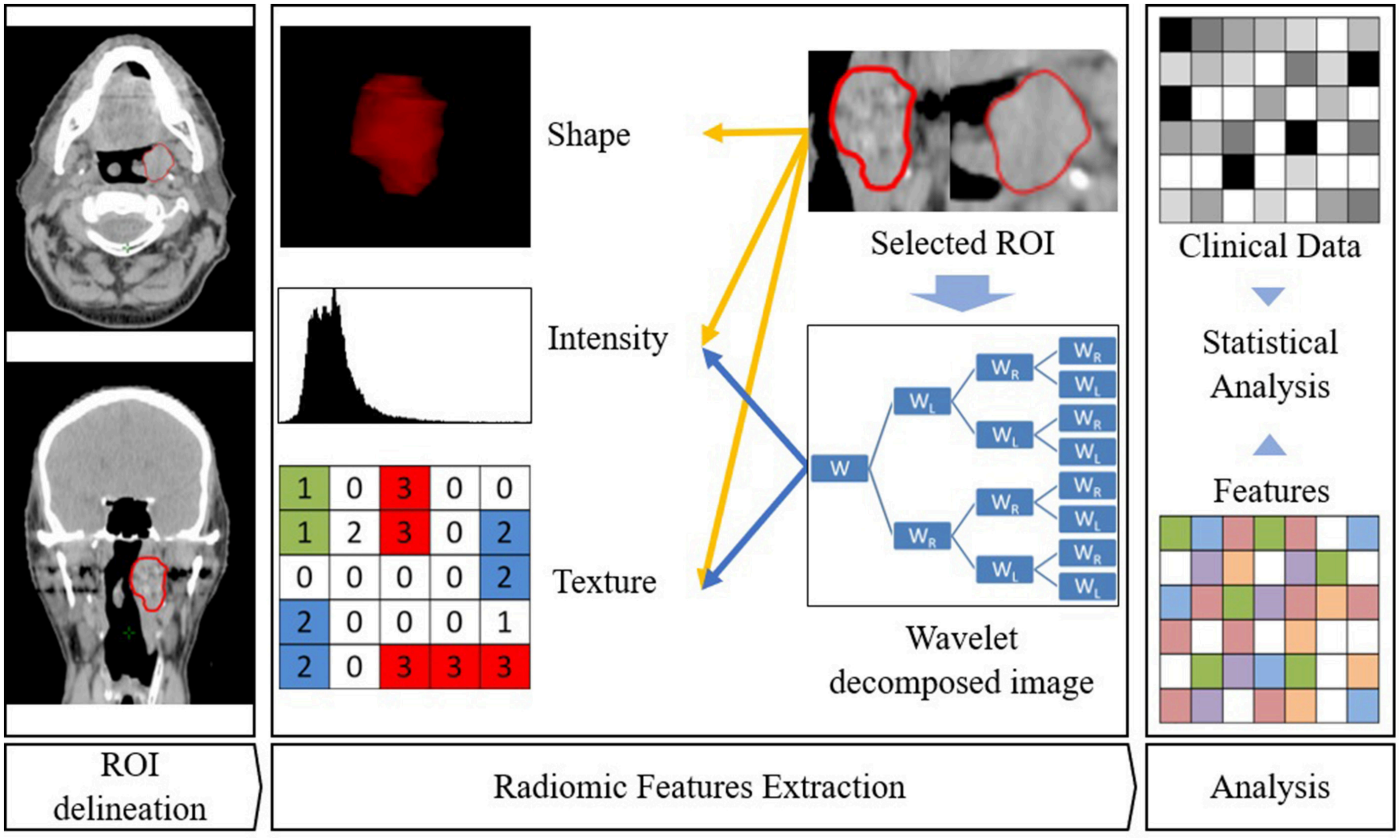
Radiomics workflow. ROI is first delineated. Then features are extracted from this ROI. Eventually, association with clinical parameters or survival are sought.

A high number of features is usually extracted and a selection needs to be made, generally by the following steps: each region/volume of interest is contoured by two independent physicians to assess feature stability: only reproducible values independent of delineation uncertainties are kept, using Intra Class Correlation (ICC). Then univariate analysis is performed to select features with a statistically significant correlation with the endpoint. Eventually multivariate analysis and feature ranking are performed. The best performing features are then combined into a predictive model. The performances of the resulting model is then assessed on an external validation dataset (Figures 1, 2). Due to the high number of interdependant features generated, ML algorithms are well-suited to render best performing models. The rise of research in radiomics has relied on the recent accelerated development of ML techniques.

The reproducibility of such a model is also highly dependent of the imaging technique (7, 8). Movement may alter the model performances as well as the time lapse between contrast injection and acquisition, or data processing from acquisition to display (from RAW images to DICOM images), known to vary across manufacturers.

Several institutions have dedicated teams for radiomics research with an open source approach. For instance, the radiomics.io plateform (http://www.radiomics.io/) was developed by Harvard University in order to develop and maintain open source projects, provide go-to resource for radiomic applications and promote radiomics within the science community. It provides links to major radiomics original articles and features extraction tools such as PyRadiomics (9). The CASMI (Chinese Academy of Science, Medical Imaging) laboratory in Beijing has developed a website on which tools such as *3DMed* (feature extraction software) and *Radiomics Software* (automated segmentation and radiomics classifier software) are available for download (http://www.radiomics.net.cn). It also launched a cancer data sharing plateform. Other features extraction softwares have also been developed and made available to the scientific community such as IBEX (10), LifeX (11).

### Machine Learning

**Figure 3:**
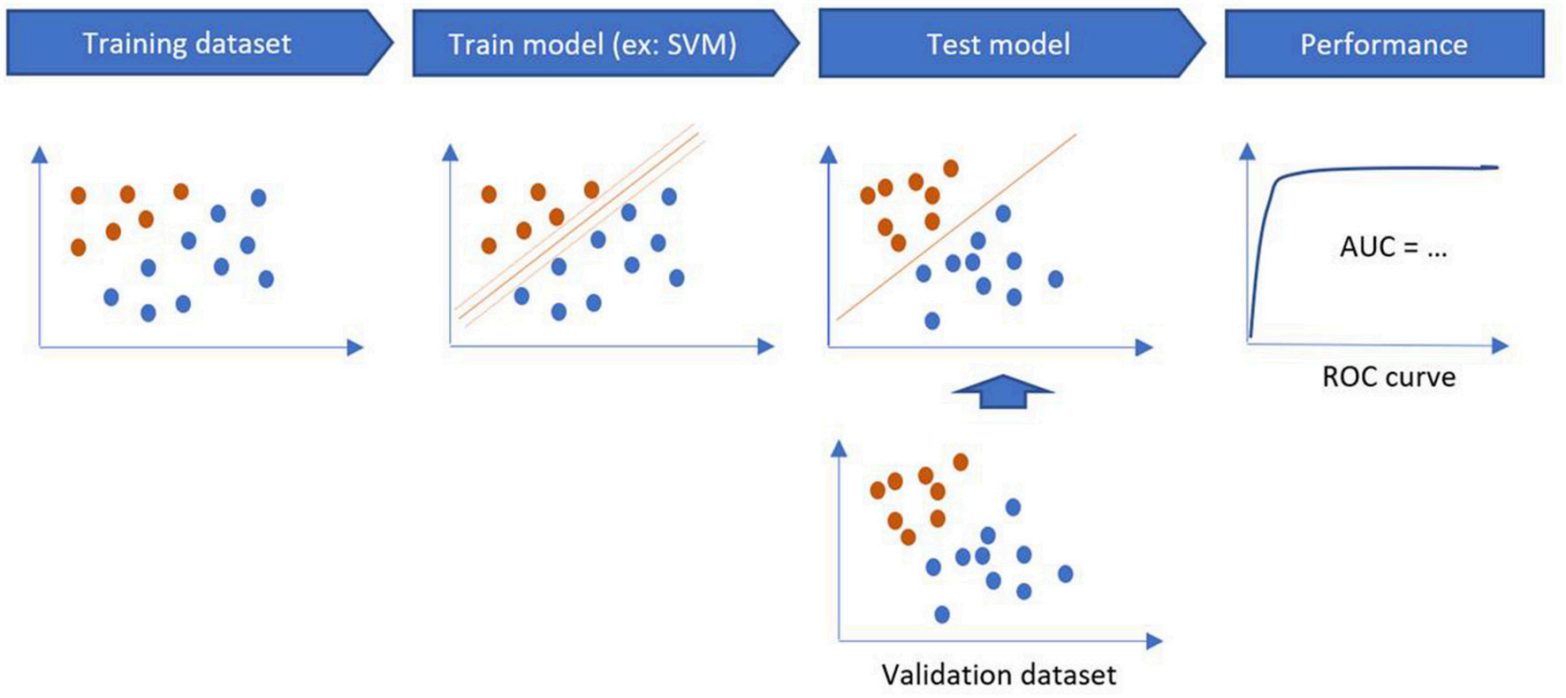
Training and validation steps of a machine learning algorithm.

ML technique is a branch of artificial intelligence in which an algorithm learns by inference from a data set (12). Its main objective is to produce a model capable of classification, prediction, and estimation of a situation from selected known data. As a result it may improve our decision-making process as it can encompass a higher number of parameters than humans (13). Parameters coming from clinical observations, biology, genomics and radiomics data may improve the clinician decision-making process (Figure 3).

The training phase is the process of finding dependencies in a system for a given dataset. Then, these dependencies are used to predict new outputs. ML methods are categorized into supervised and unsupervised learning. Supervised algorithms use labeled data (to map the data to the desired output classification) while unsupervised learning algorithms do not use labeled examples and provide a classification based on the patterns found in the dataset. Semi supervised learning methods combine supervised and unsupervised learning when dataset combines both labeled and unlabeled data.

**Figure 4:**
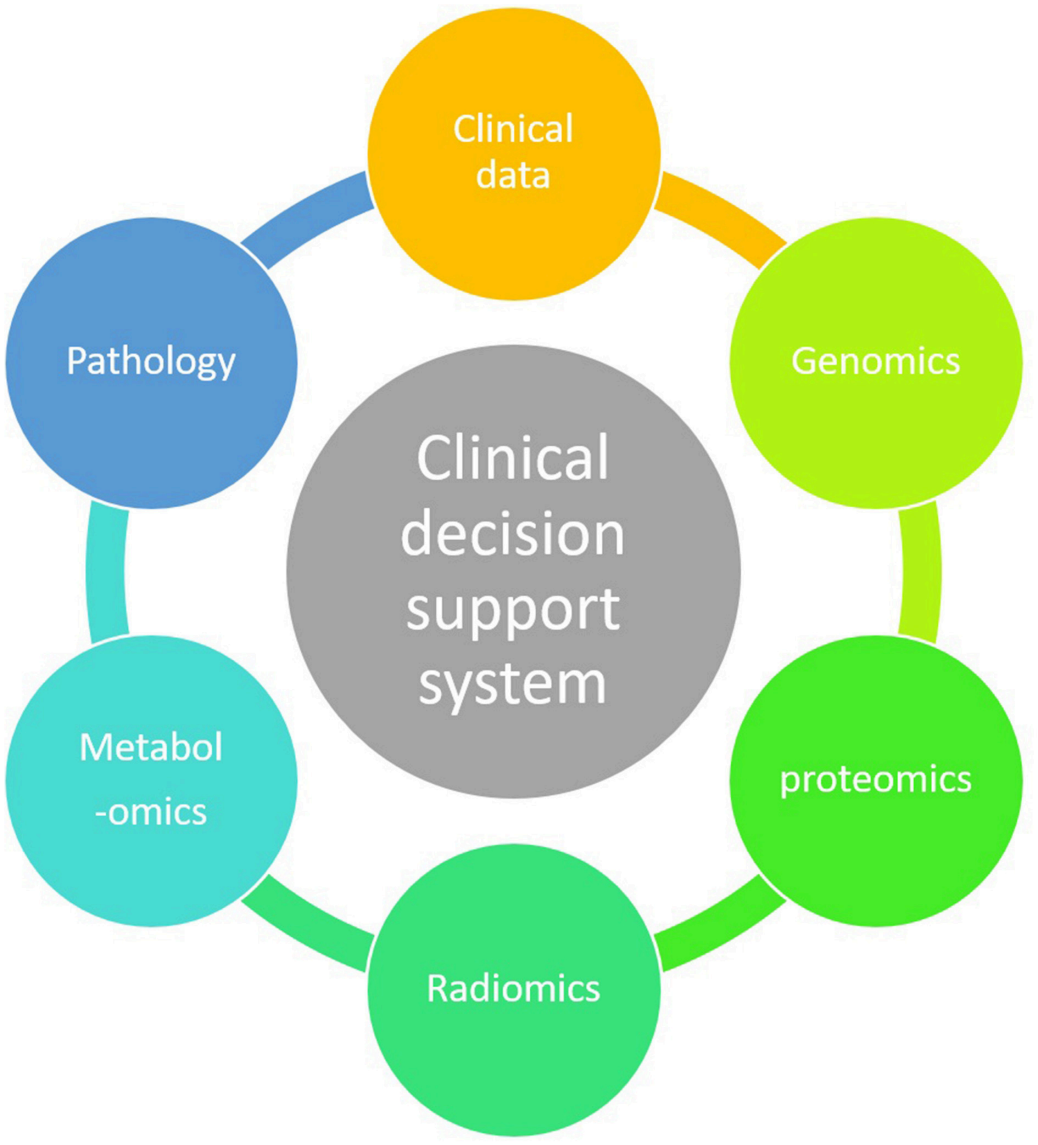
Holistic clinical decision support system.

The performance of a model is estimated on a validation sample, through sensitivity, specificity, accuracy and area under the curve (AUC) on the testing sample (Figure 4). If there is only one dataset, validation methods such as Holdout Method, Random Sampling, Cross-Validation, and Bootstrapping can be used (14).

Machine Learning methods include, but are not limited to:

- ANN (artificial neural network) can be used to classify patients. Its main disadvantage is its high level of abstraction which makes it difficult to determine what features were used by the neural network. This disadvantage is called the “black box” effect.
- DTs (Decisional Trees) renders a decisional tree to classify each input.
- Random forest classifier uses a vote from multiple decisional trees for a better classification compared to a single decisional tree.
- SVM (Support Vector Machine) technique identifies a hyperplane that separates data points in two classes. Its advantages are its generalizability and the possibility to obtain probabilistic outputs.
- BNs (Bayesian Networks) produce probability estimations that are useful for representing dependencies between input features through graphs.

Limitations of machine learning lie in data quality (missing, duplicated, noise, outliers) which is the corner stone of quality algorithms. Besides, a higher number of features (data type) implies a higher number of samples (15). Data sample should be 5–10 times more numerous than the number of features. As a result, data processing is necessary to reduce dimensionality and enhance the model robustness (16). However, for a given question such as prognosis, many signatures with different features are generated resulting in a lack of agreement on the features to take into account (17–20).

The ideal classification model should fit the training set and correctly classify each item in the test dataset. However, if misclassification error rate is low in the training set, and gets higher in the testing set, the model is overfitting and is not generalizable.

As a result, validation on external cohorts is critical to prove its generalizability. Many models (not necessarily developed with machine learning techniques) are shared on plateforms such as http://www.predictcancer.org. This open source initiative may ease external validation of models by other teams on other populations and promoted by radiomics websites previously introduced.

## Materials and Methods

### Descriptive Approach of Machine Learning Application

We performed a description of applications of machine learning at each step of treatment of head and neck cancer in radiotherapy.

### Review on Radiomics and Machine Learning Survival Models

We then focused on radiomics with a systematic review in accordance with the Preferred Reporting Items for Systematic Reviews and Meta-Analyses (PRISMA).

We conducted a comprehensive search of PubMed for relevant peer-reviewed publications from January 2012 to January 2019. A comprehensive list of MeSH terms and keywords was used to query Pubmed (“head and neck cancer,” “radiomic,” “signature,” “survival,” “machine learning”). The search strategy also included screening of reference lists of relevant publications (“snowball” search technique). Publications of models based on radiomics or machine learning to predict survival or distant metastasis in head and neck cancer after radiotherapy was performed.

## Radiation Oncology Treatment Planning

Radiotherapy treatment planning encompasses time consuming tasks such as delineation, dosimetric planification, and adaptive radiotherapy. Increasing automation of these tasks is a promising prospective. It may shorten and improve reproducibility of contouring that limits the implementation of adaptive radiation therapy and Normal Tissue Complication Probability (NTCP) modeling.

### Automatic Organ at Risk (OAR) Delineation

The delineation process is manual and time-consuming and suffers from inter and intra practitioner variability. Automatic OAR delineation may help for contouring homogenization and ease the workflow, a key factor for implementing adaptive radiotherapy. The initial approach for automatic delineation was mainly atlas based, using hybrid registration to deform structures from an atlas and map it to the patient anatomy.

Yang et al. (21) created an algorithm for automatic segmentation of the post treatment parotid glands. Fifteen patients were prospectively included. They had head and neck cancer treated by radiation therapy with at least 1 year of follow-up. Parotid glands were manually contoured on a pre-treatment MRI. This volume would then be used as an atlas for each patient. MRIs were performed at 3, 6, and 12 months after radiation therapy. The aim was to have the parotids glands automatically segmented on follow-up MRI to assess their evolution. The first step was an atlas-based registration: first, a hybrid deformable image registration was used to map the pre-RT MRI to the post-RT MRI. The resulting transformation was applied to delineated parotid volume. Multiple features were extracted for input aside with the transformed contour for the support vector machine training in order to detect horizontal, vertical and diagonal edges and strength of edges.

The trained SVM could detect edges on the follow-up MRI to render a parotid contour, then intersected with the transformed parotid volume + 10 mm. Smoothing and 3D morphology operations were then applied to render a volume without disconnection, holes or irregularities.

The ground truth was manually contoured on a follow up MRI. A volumetric Dice-Sørensen Coefficient (DSC) was performed to assess the model performance. There was no significant difference of volume between the automatically and manually contoured parotid glands on follow-up MRI with a volumetric Dice-Sørensen Coefficient of more than 90%.

However, such an atlas based method provided little improvement to the workflow since the result usually needs correction (22–31). Using artificial neural networks is a new approach: Nikolov et al. (32) recently created a convolutional neural network using a 3D U-Net architecture model (33) to automatically contour 21 OAR. The validation process had to account for human variability: each case was segmented by an experienced radiologist arbitrated by a second delineation; their contour were then compared with two further radiographers arbitrated by experienced radiation oncologists as the ground truth. The performance was assessed by a surface DSC as it provides a quantitative agreement between the two surfaces instead of the two volumes.

This assessment method is more clinically relevant since it better represents manual correction time for every misplacement. Delineated OARs were the brainstem, spinal canal, spinal cord, left and right cochleas, lacrimal glands, lenses, lungs, optic nerves, orbits, submandibular, and parotid glands. Each OAR surface DSC was similar to humans in all previously described OAR except for the right lens and the brainstem (deviation of more than 5%). Right lens uncertainties may be correlated with CT quality as borders are easier to see on a higher definition CT. Interobserver variability among experienced oncologists (for instance parotid delineation) was a limitation for learning since the ground truth was not consensual.

Surface DSC is a metric introduced by Nikolov et al. to take into account misplacement even by a small offset that may be time consuming to correct compared to volumetric DSC. This convolutional neural network approach improved the usual performance of automatic delineation model. Clinical acceptability and time saving estimations need to be assessed to value the model's clinical implementation. Integrating other imaging modalities (MRI, PET, CT) co registration to better contour certain OAR has to be assessed.

### Adaptive Radiotherapy

Intensity Modulated RadioTherapy (IMRT) is the standard of care for Head and Neck radiotherapy. Daily repositioning by kV-CBCT (Kilo-Voltage Cone Beam Computed Tomography) or MV-CBCT (Mega Voltage Cone Beam Computed Tomography) through rigid registration to detect and correct set up errors (34) can be performed. Adaptive Radiation Therapy (ART) is the adaptation of target volumes and critical OAR volumes to tumor shrinkage and patient emaciation during treatment (35–37). Benefits and feasibility of ART has been assessed by exploratory studies (38–40) ART is not routinely used, as a consequence of limited resources, its time consuming character, and unreliable automatic contouring.

Thus, the main obstacle to test ART on a higher number of patients is the supplementary workload even with hybrid deformable image registration (HDIR) (35). Guidi et al. created (41) and tested (42) a support vector machine tool for adaptive tomotherapy treatment in head and neck cancer to completely automate this process in order to raise an alert when the patient would need a new dosimetric scan to adapt the volumes. Between 2013 and 2014, 40 patients diagnosed with a head and neck cancer treated by radiation therapy with 66 Gy on high-risk volume and 54 Gy on low-risk volume were included. Raystation® (v 4.5.1.14 RaySearch Laboratories AB, Stockholm Sweden) TPS was used for HDIR. First, a rigid registration was performed and ROIs copied on the MVCT. Then, HDIR was performed and the resulting mesh grid—with a voxel to voxel map of the deformed vector field—was eventually applied to new automatically contoured ROIs. Then dose deformation was carried out using the deform dose tool of the TPS.

The algorithm classified data that included ROIs and several dose metrics on a weekly basis.

Eventually, the SVM model rendered a graph and a summary of the output. Unsupervised learning identified four categories:

- Predictive: the data points are within the defined threshold. Re-planning is not needed;
- Adaptive: Points are outside the defined threshold. The patient needs a replanification;
- Errors: points that are outside thresholds because of a software bias;
- Warning: points that are outside thresholds because of emaciation, organ motion, or incorrect set up;

After a learning phase on 27 patients, the algorithm was tested on 13 patients. In the first 3 weeks, no replanification was needed for 84% of the patients. Then a reverse trend became significant at the beginning of the 4th week with 77% of replanification needed during the last 2 weeks of treatment.

Guidi et al. then performed a clinical validation retrospective cohort of 90 patients (42) from four radiation oncology centers equipped with various accelerators (Tomotherapy or Linac + CBCT) to assess the performance of the previously described algorithm. Two physicians suggested an ideal re-planning time—point if re-planning had to be performed. Decision was based on parotid gland and neck volume shrinkage in comparison with the initial dosimetric CT scan.

Similarly to Yang et al. (21) after 6 weeks of therapy, parotid gland volume decreased by 23.7 ± 8.8%. As shown on their previous work described above (41), there was no re-planning needed during the first 3 weeks (for 86.7% of cases). From the 4th week, 55.2% of patients would need a re-planning. 11.8% were affected by biases and 5.9% would generate a warning.

Giudi et al. provide a machine learning powered, automated tool to discriminate treatments in need of replanification. The clinical validation was focused on the time point of re-planning reproducibility compared with physician decision.

Many adaptive ART approaches are being investigated and questioned (40). Dose recalculation models are based on patient cohorts and are thus limited to data availability. It should be enhanced with the rise of data mining and further validated. The machine learning approach proposed by Guidi et al. opens the prospect of triggering a clinical decision in an automated process, leaving only equivocal cases to the radiation oncologist. However, this would require an automatic workflow handling with a powerful TPS computing power.

## Normal Tissue Complication Probability Assessment

### Predictive Model Limitations

NTCP models are usually based on dose–volume histograms (DVHs), which are not ideal representations of 3D doses and assume that all the regions of an OAR have an equal functional importance, thus discarding organ specific spatial information. They do not take into account fraction dose variations, nor anatomical variations of the OAR and its dosimetric consequences during treatment (43). Models taking into account more data such as 3D dose distribution in OARs, dependencies between the dose delivered at other OARs may enhance toxicity prediction. Models using machine learning might be well-suited since the bigger amount of interdependent data to take into account.

### Xerostomia

Xerostomia is one of the most limiting toxicity during radiation therapy for head and neck cancer due to its consequences on weight loss, tooth decay, speaking difficulties, dysphagia (21, 44). A better prediction of parotid gland function would enhance its protection and the quality of life after RT. Parotid volume diminishes with radiotherapy (21, 41, 45). The volume reduction and the migration toward high dose region can generate a total absorbed dose significantly higher than the planned one. Consequently, a relation between parotid gland volume reduction and xerostomia was found (46). As previously described, Yang et al. (21) generated an atlas based model for parotid delineation. The volume tended to decrease with time after radiotherapy consistently with previous finding (44). New NTCP models predicting xerostomia using volumetric change and other parameters could be tested without the limiting burden of time-consuming contouring. Further longitudinal studies using this algorithm may investigate parotid gland volume reduction as a predictive factor of xerostomia.

Zhang et al. published a model for a plan related clinical complication probability in a multiplan IMRT framework (47). They aimed to predict a given OAR complication as a function of all OAR dose volume constraint settings without explicit additional computation. Using machine learning, such a prediction may be possible. One hundred and twenty-five plans for one head and neck by varying Dose Volume (DV) constraints on right or left parotid, or cord were created

The sample was randomly partitioned between training and testing samples with a 50-time cross validation. Decision trees and support vector machine were explored. SVM yielded superior results in predicting saliva flow rate and decision trees rectal bleeding.

The ground truth on salivary flow estimated by an Equivalent Uniform Dose based model developed by Chao et al. (48) trained on retrospective studies (48–50). Compared to this model, plan related post RT salivary flow rate prediction using SVM had a 0.42% (0.41–0.43) error.

This multiplan approach to display the risk of an OAR adverse event regarding dosimetry constraints on other OAR is global and may help physicians in their prescription. The ground truth relies on relatively weak and somewhat heterogenous data (retrospective cohorts) that may bias the prediction tool, but on the other hand, it easily integrates these data in the clinical decision. This approach would now require a clinical validation.

### Mucositis

Mucositis is commonly seen in head and neck radiation therapy treatments, leading to anorexia, pain, dysphagia, weight loss (51), reduced quality of life (52) and eventually missed fractions (53), higher treatment duration compromising tumor control (54). Mucositis limits dose escalation or hyper fractionation designed for better oncology outcomes (55).

Many NTCP models have been developed for clinical decision support, treatment modality selection, and treatment plan optimization (56–59). However, none can predict mucositis severity and guide clinical decision. This may be due to their DVH-based nature, leading to oversimplification of the dose distribution [since it was previously shown that xerostomia was impacted by spatial distribution of the dose (60)]. Oral cavity contains keratinised and non-keratinised mucosa, thus DVH based decision may be biased. Dean et al. conducted a study to generate a severe acute mucositis NTCP and provide with the generated model clinical decision guidance, based on dose constraints (61).

Three hundred and fifty-one patients enrolled in six prospective trials were included. Patients had been treated for various head and neck cancers and had DICOM RT files available. Mucositis was graded with the common terminology criteria for adverse events CTCAE v2 or 3 which are almost equivalent. Mucositis was dichotomized between severe (grade 3 and more) and non-severe (grade 2 or less). Induction chemotherapy, concurrent chemotherapy regimen, definitive vs. post-operative radiotherapy, primary disease site, age, sex and relative cumulative dose–volume histogram in 20 cGy intervals from 20 to 260 cGy per fraction were co variates of the models. Statistical analysis used for ML methods followed the principles described by Kang et al. (15) for model generation, and on the TRIPOD guidelines (Transparent Reporting of multivariable Prediction model for Individual Prognosis or Diagnosis) (62). Three different types of classifiers were generated: penalized logistic regression (PLR) (63), support vector classification (SVC) (64) and random forest classification (RFC) (65). They had approximately the same discriminative abilities. The addition of the dose distribution description did not improve the discriminative abilities. Therefore, the standard models (without the spatial dosimetric data) were favored. The standard RFC model had better calibration, probability estimates and overall performance than PLR and was the easiest to understand. As a result, the RFC model was preferred for prediction of severe mucositis for individual patient. It is available online at https://github.com/jamiedean/oral-mucositis-model.

The volume which received 220 cGy or more by fraction (V220) was the most important feature in both RFC models. The higher fractional dose volume (v240 and v260) were close to 0% and therefore did not correlate with mucositis. Age was the clinical covariate of highest importance but this may be artifactual due to the large number of possible values compared with the other clinical co variates. Besides, on univariate logistic regression, it was not significantly associated with severe mucositis.

Another model was published (66) with a similar findings on dose response (volume of oral mucosa receiving more than 2 Gy per daily fraction was the most strongly associated feature). Concurrent chemotherapy was also correlated contrary to the Dean et al. model, perhaps since the V220 cGy volume was positively correlated with concurrent chemotherapy. The Dean et al. model was based on delineation standard that does not accurately represent the mucosal surface and may explain the lack of improvement with spatial dosimetric data. Dean et al. recently created and validated a novel automated contouring technique for oral mucosa that would be more reproducible and representative of the mucosal surface (67, 68). Data on tobacco and alcohol consumption were not available for patients in two out of six trials.

## Tumor Control Probability Assessment

Improving the tumor control probability assessment before treatment is a promising way to adapt the treatment strategy. Head and Neck tumor treatment strategy mainly depend on the TNM stage. Radiomics are correlated with tumor characteristics, which may provide improved prognosis classification.

### Radiomics Revealing Tumor Characteristics

#### HPV Status

Yu et al. (69) developed a model to assess HPV status with a radiomic signature: they extracted 1,683 features with IBEX (10) from head and neck GTV T or N (or both, in this case the most extreme value was used). Feature selection was carried out in a stepwise manner: (1) inhomogeneous features between training and validation cohorts were discarded; (2) only features which could discriminate HPV + and – were kept; (3) only biserial absolute correlation > 0.3 between each radiomic feature and HPV status were kept (4) only the top 10 features regarding their mean AUC (assessed by various statistical models) were kept (5) final features were selected by forward selection (one by one add to the model until the model AUC ceases to increase).

The General Linear Model (GLM) was used as it provided better AUC compared with various other models (such as random forest, SVM, decision trees, deep learning…). MeanBreadth and Spherical Disproportion were the most important features. MeanBreadth measures width of the ROI, thus it is correlated to tumor size. SphericalDisproportion represents the complication of the surface compared to a sphere. This may imply that HPV + tumors are smaller and less complex compared to HPV—tumors. The model performance was tested on two dataset coming from a public and a private hospital. The model performed an AUC of 0.86667 on public leaderboard, and 0.91549 on the private one which is a good indicator of generalizability. Clinical data such as grade or TNM stage although available, were not used as authors wanted to assess the image discriminatory power, and the discretization induced by these variables could add uncertainty to the final model.

#### Extra Nodal Extension

Nodal metastasis and tumor extra nodal extension (ENE) are key decision factors for cancer management. However, the performance for ENE detection by imaging measured by AUC range between 0.65 and 0.69 (70). From CT scans of 270 patients from 11 centers, Kann et al. (71) developed a convolutional neural network (CNN) trained on 2875 lymph nodes delineated and labeled with histologic features. After cross validation on 124 samples, the CNN was then tested on 131 samples. To avoid overfitting, data augmentation by random rotations and flips was used. Three different networks were used (Boxnet, SmallNet, DualNet). Dualnet was selected for further testing as it yielded the best AUC. On independent test set, the DualNet neural network demonstrated an AUC of 0.91 (95%CI: 0.85–0.97) with a negative prediction value (NPV) of 0.95 for ENE status, and a AUC of 0.91 with a NPV of 0.82 for nodal metastasis. Using a convolutional neural network directly on images, without prior features extraction, on raw DICOM is a robust approach as it frees from the variability of radiomics features and image pre-processing. Furthermore, they performed a feature extraction with RF analyse with lower performance compared to convolutional neural network. Only nodes that could definitively be correlated with histologic reports were included, which may lead to a selection bias. CT scan parameters heterogeneity was minimized by normalization and scan resampling.

### Machine Learning on Clinical Factors for Prognosis Assessment

Bryce et al. (72) developed an artificial neural network on clinical factors and compared it to a logistic regression approach to create a predictive model of the 2-year recurrence free survival. They analyzed data from a phase III trial (73) that included patients with locally advanced head and neck squamous cell carcinoma (≥T3 or N3 or T2N0 base of tongue, hypopharyngeal, or pyriform sinus carcinomas) undergoing hyperfractionated accelerated radiation therapy with or without concurrent cisplatin and 5FU. Of the 116 patients of the study, 95 were included. Fourteen clinical variable potentially associated with 2 year PFS (Progression Free Survival) were available. Initial univariate analysis followed by stepwise multivariate analysis were first performed to select a subset of promising variables. This subset was pruned through stepwise elimination from an ANN model. The neural network used was a three layers feed forward back propagation neural network (74). For logistic regression models, clinical variables were first selected by stepwise selection and backward elimination with an assessment of the model performance to confirm that the selected variables were the best for 2-year survival prediction. The best logistic regression model used nodal stage, tumor size and race with an AUC of 0.67 ± 0.5. At 70% sensitivity, LR performed a 54% specificity while artificial neural network performed a higher AUC of 0.78 ± 0.05 (*p* = 0.07) with a specificity of 72% for a 70% sensitivity. As a result, ANN performed a better model of the 2-year survival. Although its performance remains low for a clinical use, it was a sensible improvement in prediction compared to TNM alone or logistic regression model within the limits of the available data (≥T3 or N3 or T2N0 base of tongue, hypopharyngeal or pyriform sinus carcinomas). As a result, the 2-year follow-up was too short to reveal the effects of chemotherapy. Due to this non-linear and non-parametric superior ability to model complex patterns, ANN techniques provide a new approach for predictive medicine, but rely on the quality and quantity of data available and its performance is always limited to characteristics of the patients in the training set.

### Radiomic Signature Added Value

Radiomic features can predict tumor characteristics linked to survival. But do they provide additional information for a better prognosis classification? Ou et al. (75) developed a radiomic signature to estimate overall survival and assess its incremental value to HPV status. One hundred and twenty patients with stage III—IVb (TNM 2010) were included from 2006 to 2010 in a former monocentric study cohort (76). They received either cetuximab (BRT group) or cisplatin (CRT group) concurrently with a 3 dose levels radiotherapy (70, 60, 50–54 Gy). Oncoradiomics™ was used to extract 544 features, narrowed to 24 statistically significant features by logistic regression, from which a radiomic signature score was generated. On the whole population, the signature score had prediction capacity of 5-year survival with AUC = 0.67 95% CI [0.58–0.76]. The fraction of patient whom HPV status was unknown in BRT or CRT groups was not detailed. P16 status also predicted for OS (Overall Survival). Patients with low score had significantly better OS and PFS. When combining p16 and radiomics signature the prognostic performance improved (AUC = 0.78, *p* = 0.01). This preliminary study showed an incremental added value of a radiomic signature score to HPV status. It would require external validation on a more homogenous population as treatment standards evolved between 2006 and 2012.

Radiomic signature reveals a tumor phenotype that may not be organ specific. For instance, Aerts et al. (6) generated a four feature radiomic signature on a retrospective cohort of 422 patients diagnosed with Non-Small Cell Lung Cancer (NSCLC) treated with curative intent. The signature's validation was performed on a 225 NSCLC patients cohort, two Head and Neck Squamous Cell Carcinoma (HNSCC) patients cohort, and a 89 patients HNSCC cohort with genomic information. The four feature radiomics signature was validated using the concordance index [c-index or CI, a generalization of the ROC AUC (77)]. On Lung2 data, performance was good with a CI of 0.65, *p* = 2.91.10^−9^ (Wilcoxon test). On H&N1 and H&N2, performance was high with respective CI of 0.69, *P* = 7.99 × 10^−7^ (Wilcoxon test), and CI = 0.69, *P* = 3.53 × 10^−6^ (Wilcoxon test).

Compared to volume, the radiomic performed significantly better. When combined to TNM classification, the performance was significantly better than TNM alone suggesting complementary information for prognosis. These performances were improved both in the HNSCC and the NSCLC cohorts, in all the treatment groups (radiation or concurrent chemo radiation). This signature was not correlated with HPV status and did preserve its performance in the HPV—group.

Aerts et al. showed the predictive value of features selected for their stability and reproducibility which indicates the power of integrating independent datasets for feature selection and model building. Among those features, keeping only the top four performing features regarding prognosis to perform a single validation on 545 patients allowed a robust statistical analyse and avoided overfitting. The most dominant features of the signature quantified intra-tumor heterogeneity. Intra-tumoral heterogeneity reveals the presence of multiple colonies with different gene expression that cannot be encompassed by biopsies and therefore is an obstacle to personalized medicine and a potential prognosis marker. This results could be supported by associations with gene expression found by Zhu et al. on the TCGA and TCIA cohorts (78). The better prognosis prediction performance in head and neck cancer may be due to the absence of breathing movement but underlines the non-specificity of heterogeneity of this signature in cancer prognosis.

The MD Anderson Cancer Center head and neck quantitative imaging working group (79) has developed a radiomic signature on 465 patients with oropharyngeal cancer to assess the recurrence probability after IMRT. Patients were split into training, tuning and validation sets. For each GTVp, 134 radiomic features were extracted using IBEX and narrowed to two features with decision tree modeling: Intensity Direct Local Range Max (LRM) and Neighbor Intensity Difference 2.5 Complexity with cut-off values corresponding to 1,616 and 457,808, respectively. Patients with both values were below the aforementioned cut offs, had a more favorable local tumor control with 94% of tumor control rate at 5 years in the training set compared to 62 to 80% depending if one or two values were above cut-offs. Compared to clinical factors such as HPV, smoking status or age, the radiomic signature performed better. Limitations come from CT heterogeneity and some missing clinical data (HPV status, tabaco consumption status).

Nasopharyngeal carcinomas 5-year overall survival is around 50% (80), but drops when in advanced stage. Prediction of outcome may help to make decision regarding treatment. Zhang et al. (81) developed a predictive model for 3-year PFS using MRI-based radiomics in nasopharyngeal carcinomas. One hundred and eighteen patients were included. Nine hundred and seventy features were extracted using Matlab. A radiomics signature from 8 features (5 from T1 weighted and 3 from T2 weighted images) yielded a C index of 0.737 (95% CI: 0.549–0.924). This performance outperformed classic TNM staging system (C-index: 0.514), but both combined yielded a better performance (C-index: 0.761; 95% CI, 0.664–0.858) with a good calibration.

Being able to predict not only the risk of recurrence but also whether it would be locoregional or a distant metastasis (DM) may enhance treatment decisions. As a result, Li et al. (82) explored the predictive power of radiomics for the type of recurrence (in field, out of field, marginal). After selection of the influential subset of radiomic features, Kruskal Wallis test and ROC analysis were employed for each feature to assess its capability on in field recurrence prediction. After further selection, several machine learning models were trained (Artificial Neural Network, K Nearest Neighbor and Support Vector Machine). Three hundred and six patients were included with a median follow up of 26.5 months. Eighteen in field, one marginal and one out of field recurrences occurred. Patients with in field recurrence could be differentiated by 8 features. The best performing model was artificial neural network (AUC: 0.812).

Zhang et al. (83) developed a MRI based model for distant metastasis (DM) prediction in nasopharyngeal carcinoma. DM is a common cause of treatment failure in nasopharyngeal carcinoma. One hundred and seventy-six patients from a retrospective multicentric cohort were included, 123 in the training cohort and 53 in the validation cohort. Pre-treatment MRIs, clinical and biological data were gathered. The primary endpoint was the time to primary MRI to DM or censoring. The follow up ranged from 36 to 60 months after the primary MRI. All patients received concurrent platinum based chemoradiotherapy. MRI were performed with various protocols, thus intensity images were normalized using PyRadiomics plateform. Two senior radiologists segmented images blinded from each other to assess interobserver reproducibility. Two thousand eight hundred and three imaging features were extracted using PyRadiomics platform. Feature selection included many steps: First interclass correlation coefficient (ICC) was calculated to assess the effect of segmentation variability, only features with an ICC above 0.75 were kept. Then, univariate analysis for survival using Chi^2^ or Mann–Whitney *U*-tests were performed for each features. Only statistically significant parameters were kept (*p* < 0.05). Then minimum redundancy maximum relevance model was applied to keep the top 10% features. Eventually, the most significant features with highest area under the curve were selected using LASSO (Least Absolute Shrinkage and Selection operator) algorithm. A logistic regression based model to assess the DM risk was then trained. Kaplan Meier survival curves of the two groups were compared using log rank tests. The predictive ability of the model was assessed with AUC. A Distant Metastasis MRI bases Model (DMMM) was created from 7 selected radiomics features with an AUC of 0.827. In the training cohort and 0.792 in the validation cohort. Patients with a high risk of DM had a 5-year overall survival of 12% vs. 26% in the low risk group. The DM related feature with the maximum significance was “CET1-w_wavelet.HLL_ GLCM_Correlation.” It reflects the intra tumor heterogeneity from the gray scale extension perspective. The worse performance in the validation cohort suggested overfitting and further training on larger multicentric dataset should be considered. Still, being able to predict the risk of distant metastasis, or in field recurrence are promising prospective for radiomics support for clinical decisions.

Kwan et al. (84) performed a study with a similar goal on HPV related oropharyngeal carcinoma. Three hundred patients with HPV related oropharyngeal carcinoma were included. The 4 features radiomics features form Aerts et al. (6) previously described study were used. The best performing model was a combination of the radiomic signature with clinical data. In this cohort, the signature had better performance for prognostication of DM than of 5 years overall survival while it was initially designed for overall survival. The radiomics signature stratified patients for DM risks especially for cohorts with greater risks such as stage III patients.

## Discussion

Radiomics is a new way to mine data from imaging. It may provide specific information on tumoral heterogeneity, HPV status, lymphocytic infiltration and consequently, prognostic signatures.

For instance, as shown with Aerts et al. (6) gray level non-uniformity revealing intra tumoral heterogeneity was found as a bad prognosis factor which is congruent with literature and may reveal multiple clonal populations with a variable response pattern to treatments. Radiomics provide whole tumor information compared to biopsies and thus provide additional information that may become useful for treatment strategy.

As shown by Berenguer et al. (7), many radiomics features may be redundant or not reproducible. Using standardized imaging acquisition with homogenized contrast enhancement delay before acquisition, may enhance the quality of the data. Still, if all CT parameters were fixed except FOV, tube voltage, and milliamperage, then the information provided by radiomics could be summarized in only ten radiomics factors. On the other hand, too restrictive protocols may decrease the applicability to routine clinical care, all the more that with a given delay to acquisition, the actual enhancement varies depending on subjects. Another approach would be to gather CT scans with varying parameters to rule out noise in the dataset [as done by Aerts et al. (6), presented previously]. The extracted features also depend on the extraction software, patient motion and image treatment. This may be one of the reasons for variability of the generated signatures between each study (Table 1). This creates a real challenge for its implementation in daily clinical care, the ultimate goal of radiomics.

**Table 1:** Overview of radiomics based outcome prediction models.

**Study**	**Features**	**Extraction software (ES) and statistical method (SM)**	**Outcome prediction (OP) and performance (P)**	**Number of patients**	**Tumor characteristics**
Yu et al. (64)	MeanBreadth Spherical disproportion	ES: IBEX SM: GLM	HPV status AUC: 0.86667 and 0.91549	315 pts	Oropharyngeal cancers
Ou et al. (71)	24 features signature	ES: Oncoradiomics SM: Logistical regression	5 y survival P: AUC = 0.67 CI (0.58–0.76)	120 pts	stage III – IVb Head and Neck cancer from (71)
Aerts et al. (4)	Statistics Energy Shape compactness 2 Grey level non uniformity Run length non-uniformity	ES: IBEX SM: Logistic regression	Overall Survival C-index: 0.69	545 pts	Lung and head and neck cancers
Anderson (79)	Intensity direct local range max Neighbour intensity difference 2.5 complexity	ES: IBEX SM: Decision Tree model	5 y Tumour control classifier (3 groups)	465 pts	Oropharyngeal cancers
Kann et al. (67)	No prior extraction for the selected model	ES: PyRadiomics SM: Random Forest and CNN	Extra nodal extension AUC = 0.91 (95%CI:0.85–0.97). NPV: 0.95	270 pts	Nodal invasion in resected head and neck cancer
Zhang et al. (81)	8 features signature	ES: Matlab: MRI based features. SM: LASSO and Rad-Score	3 y PFS C-index: 0.737 (95% CI: 0.549–0.924)	118 pts	Nasopharyngeal carcinomas
Li et al. (82)	8 features signature	ES: PyRadiomics on SPAIR T2W MRI SM: ANN	In field recurrence Accuracy: 0.812	306 pts	Nasopharyngeal carcinomas
Zhang et al. (83)	7 features signature	ES: PyRadiomics MRI based SM: Logistic regression	Distant Metastatic MRI based Model AUC : 0.827	176 pts	Nasopharyngeal carcinomas

*GLM, General Linear model; CNN, Convolutional Neural Network; LASSO, Least Absolute Shrinkage and Selection operator; SPAIR T2W, spectral attenuated inversion-recovery T2-weighted; ANN, Artificial Neural Netwo*.

Machine learning encompasses many statistical tools suited for complex patterns detection with inter dependent and non-linear parameters. Machine learning models provide a promising enhancement of predictive model performances, suited for the increasing number of parameters available for analysis with the rise of omics. However, one of its main limitations is overfitting, leading to a lack of generalizability to other samples. To avoid overfitting, the number of patients should be five to ten times higher than the number of features (85), which requires a thorough pre-selection of features to keep the number of patients to include reasonable.

Models are only applicable on the range of data used for its generation and dependant on primary data quality. Ground truth definition is fundamental for successful model learning. It is usually defined by experienced practitioner's data labeling. In non-consensual situation, such as delineation, learning may be impaired by inter-practitioners' non-reproducibility. In this case, an approach based on comparing the model's uncertainty with the expert's uncertainty seems valid, as long as a clinically relevant metric is used. Last, each problem has its variables, and no type of machine learning algorithm can always prevail. For instance, logistic regression was better for the study of Ou et al. (75) while ANN was better suited for the clinical prediction of survival by Bryce et al. (72), which underlines the model benchmarking approach, where several models should be tested, and the best performing one kept.

## Conclusion

Potential applications of machine learning and radiomics methods in the field of head and neck cancer have been explored in several studies. Treatment planning tasks such as OAR delineation or dose recalculation are time consuming and could eventually be automated, opening the possibility for adaptive radiotherapy. Machine learning and radiomics can provide better modeling tools both for adverse events and survival for a step toward personalized and predictive medicine. While these studies provided interesting results, none of them are actually being used in the daily workflow of radiation therapy departments. Before we can reach this goal, they must be thoroughly assessed in prospective, multicentric trials to prove their actual benefit. Collaborating groups will have an important role in the design and conduct of these important studies. The use of interoperable standards and homogeneous treatment planning methods will also be needed before such study can be performed.

## Author Contributions

PaG wrote the manuscript. J-EB helped for article selection and manuscript redaction. J-EB, AB, and REA helped for technical review of the manuscript for machine learning and radiomics aspects. J-PF performed a technical review of the manuscript on radiomics and clinical aspects. AG performed a technical review of the manuscript on machine learning aspects. SK, FH, CD, and PhG performed a review of the manuscript on the clinical aspects.

### Conflict of Interest Statement

The authors declare that the research was conducted in the absence of any commercial or financial relationships that could be construed as a potential conflict of interest.

## Abbreviations

ML: Machine Learning
ANN: Artificial Neural Network
DT: Decisional Tree
SVM: Support Vector Machine
BN: Bayesian Network
NTCP: Normal Tissue Complication Probability
RT: Radiotherapy
DSC: Dice-Sørensen Coefficient
OAR: Organ at Risk
IMRT: Intensity Modulated RadioTherapy
kV-CBCT: Kilo-Voltage Cone Beam Computed Tomography
MV-CBCT: Mega Voltage Cone Beam Computed Tomography
ART: Adaptive Radiation Therapy
HDIR: hybrid deformable image registration
TPS: Treatment Planning System
ROI: Region Of Interest
DVH: Dose Volume Histogram
RFC: Random Forest Classification
GTV: Gross Tumor Volume
ENE: extra nodal extension
FOV: Field Of View.
